# Developing a new digital vision for rangeland grazing systems

**DOI:** 10.1093/af/vfag009

**Published:** 2026-02-26

**Authors:** Claire Morgan-Davies, Nicola Lambe, John P Holland, Ann McLaren, Davy McCracken

**Affiliations:** SRUC School of Natural and Social Sciences, Hill & Mountain Research Centre, Crianlarich, Scotland, FK20 8RU, United Kingdom; SRUC School of Natural and Social Sciences, Hill & Mountain Research Centre, Crianlarich, Scotland, FK20 8RU, United Kingdom; SRUC School of Natural and Social Sciences, Hill & Mountain Research Centre, Crianlarich, Scotland, FK20 8RU, United Kingdom

**Keywords:** digital hub, environment, livestock, rangeland, technology

Implications

- Identifying appropriate digital technologies is crucial to help address rangeland agricultural challenges
- A rangeland farm hub can help highlight how best to integrate environmental management into rangeland livestock systems
- A rangeland farm hub allows the development of approaches and metrics to quantify the benefits of implementing agricultural and environmental solutions
- A digital vision may increase the socio-ecological viability of rangeland livestock systems and support their resilience to ongoing climate change

## Introduction

Globally, rangeland grazing systems account for the majority of grazing lands (Reid et al., 2008) and more than half of the total land, with over 200 million people raising livestock in pastoral and agro-pastoral grazing systems. In Europe, a large proportion (59%) of these lands are classed as less favored areas (LFA—Article 2 of EU Council Directive No. 75/268/EEC) or areas of natural constraints [Article 32 of Regulation (EU) No. 1305/2013]. Although the primary function of many of these areas is to produce food and fiber, they are also rich in natural and semi-natural vegetation and support species and habitats, often with high conservation value, whose persistence is totally or partially dependent on the maintenance of specific low-intensity farming systems (Lomba et al., 2023). Moreover, across Europe, many rangeland areas are currently under pressure from biophysical and socio-economic factors, alongside broader political and cultural changes (Nori and Scoones, 2023).

These areas are also coming under pressure regarding efficiency and adaptation to climate change (Muñoz-Ulecia et al., 2024). In Europe, the EU Commission aims to be climate-neutral by 2050—an economy with net-zero greenhouse gas emissions. This objective is at the heart of the European Green Deal (European Commission, 2019) and is a legally binding target thanks to the European Climate Law (European Commission, 2025). In Scotland, the Scottish Government commitment to reduce greenhouse gas emissions to net-zero by 2045 (Scottish Government, 2024) will have a major impact on future agricultural and environmental support policies. There will be an even greater emphasis on encouraging farmers in extensive and rangeland grazing areas to improve the cost-effectiveness of their production systems and improve soil carbon sequestration [e.g. through grassland and grazing management practices, increasing plant species diversity in swards, restoring peatlands, etc. (Strack et al., 2022; Norderhaug et al., 2023)]. Scotland has also been in a biodiversity crisis (Scottish Executive, 2004) for just as long, if not longer, than we have been in a climate emergency (Cunningham, 2019). Although actions taken to address climate challenges may also have biodiversity benefits, there will be a continuing need for more targeted actions to assist other wildlife, such as upland birds that are in marked decline (Alba et al., 2022). It is, therefore, important to develop new approaches that can address both farming and biodiversity issues in these rangeland areas.

A report from the European Innovation Partnership for Agricultural Productivity & Sustainability (EIP-AGRI, 2016) concluded that the use of innovative technologies and management techniques had an important role to play, with facilitating technological innovation being an essential part of a wider array of measures required to improve the future viability of rangeland grazing systems. However, uptake of innovative technologies can be challenging in these types of systems, despite the potential benefits that their use could bring regarding farm efficiency and labor savings (Morgan-Davies et al., 2018). Developing a new digital vision for rangeland grazing systems could help address these challenges, and demonstrate the advantages of technology for farming, net-zero commitments and biodiversity benefits.

This paper showcases the use of a Rangeland Digital Hub in Scotland to develop a digital vision for rangeland grazing systems, by investigating both short-term innovation and medium to long term diversification, including land use change, focusing both on technological solutions as well as the financial, societal, and policy changes required to drive these changes.

## A Demonstration Farm Doubling as a Rangeland Digital Hub—The Case of Scotland Rural College Kirkton & Auchtertyre Research Farm

**Figure 1.**
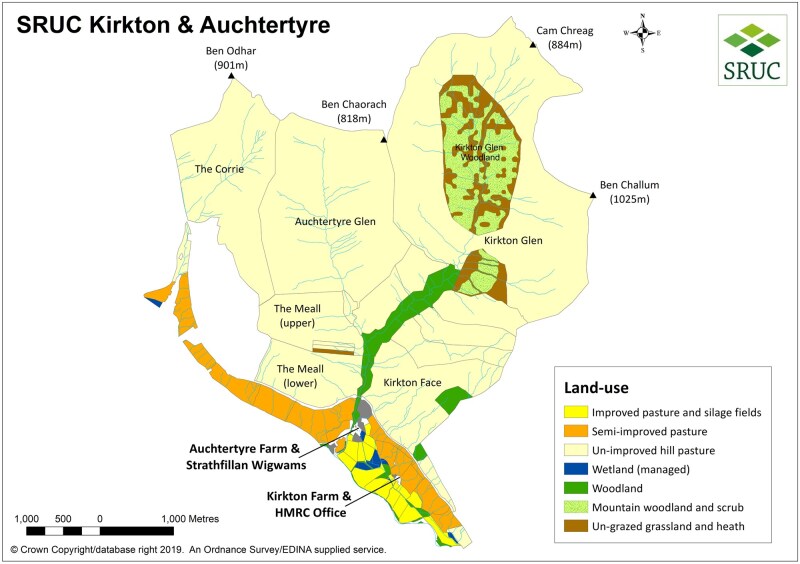
Map of SRUC Kirkton & Auchtertyre farm.

Scotland Rural College (SRUC) Kirkton & Auchtertyre mountain research farm is quite distinctive as a rangeland farm hub, i.e., a site designed to connect agricultural and environmental science with practical, real-world application and which acts as a focal point for testing, validating, and demonstrating new technologies, sustainable practices and innovative systems of direct relevance to rangelands and rangeland managers. The farm is unique due to its size (c. 2,200 ha), altitudinal range (from 170 m to over 1,000 m), and wide range of grassland, heathland, woodland, and montane habitats characteristic of mountain (hill) farming (Figure 1). It is also uniquely relevant for Scottish agriculture—of the 6.2 million hectares used for agriculture in Scotland, around 4 million hectares is rough grazing, not able to be improved by mechanical means. Hence, the outputs and knowledge created by the Kirkton & Auchtertyre hub are applicable to over 65% of the total area farmed in Scotland, and beyond.

The farm has been used as an agricultural research hub for over 50 years and as an environmental research hub for over 25 years; and is in the process of further developing its potential as an agro-forestry research hub. An overview of the wide range of national and international research currently being conducted on or associated with the farms can be viewed here (https://muckrack.com/davy-mccracken-1/portfolio), drawn from press articles and Farm Advisory Service material aimed at upland land managers.

SRUC Kirkton & Auchtertyre farm has been part of the Global Farm Platform Initiative (GFP) since 2018. Established in 2014 and honored this year by the FAO Technical Recognition for Sustainable Livestock Transformation (FAO, 2025), the Global Farm Platform Initiative is a collaborative network of 19 research farms and 28 institutions across six continents, dedicated to optimizing livestock production whilst minimizing environmental impact. Through its hub-and-spoke model, where research farms (hubs) serve as regional centers for experimentation and demonstration, while commercial farms and smallholders (spokes) adopt and disseminate locally relevant, economically viable, and environmentally sustainable practices, the GFP integrates cutting-edge science with real-world farming, fostering sustainable livestock transformation, One Health, and global food security (www.globalfarmplatform.org). As a major partner in this network, SRUC has sought to establish the Kirkton & Auchtertyre research farm as an innovative world class resource for addressing 21st century rangeland land use challenges and opportunities.

## Developing a Digital Vision

**Figure 2.**
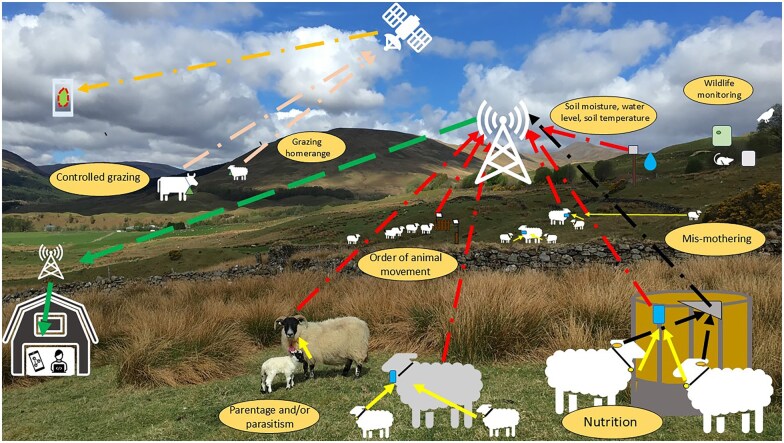
Schematic representation of the Rangeland Digital Hub with examples of associated research activities.

Our approach (Figure 2) is based around the establishment of a unique *Rangeland Digital Hub* and a program of associated research and demonstration activities. Themes within this program include:

- Moving toward a Digital Twinning concept (Purcell and Neubauer, 2023) with real time data capture including Internet of Things (IoT) technologies;
- Rangeland livestock systems fit for the 21st century;
- Optimizing the integration of environmental management into rangeland farms;
- Increasing the socio-ecological viability of rangeland farming;
- Development of natural capital assessment metrics;
- Knowledge exchange for impact.

### A rangeland digital hub and moving toward a digital twin

**Figure 3.**
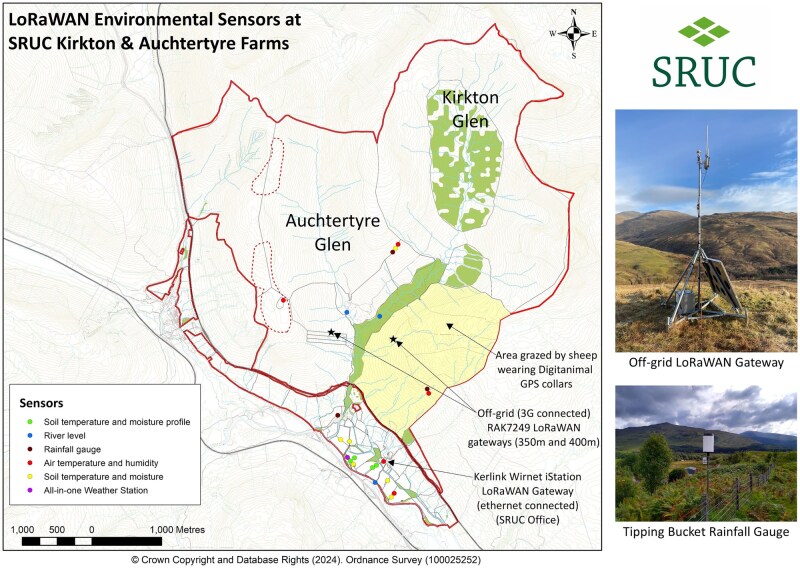
Map of the environmental sensors’ locations at SRUC Kirkton & Auchtertyre.

Over the past nine years, the first remote, upland IoT (Internet of Things) low-power wide area network (LPWAN) in the UK has been established on the Kirkton & Auchtertyre farm (McCracken, 2017). It is used to collect near-real-time data from sensors on livestock (assessing, e.g. location, health, and welfare variables) and in the wider environment (measuring, e.g. soil temperature, moisture content, river levels, meteorological data; Figures 2 and 3). The intent is to further develop the use of sensors and associated technologies within the Rangeland Digital Hub to assess and demonstrate the benefits to be gained from not only having access to such otherwise difficult to collect data streams but also from being able to combine and interpret the information coming from the different data streams. This role of interpretation is becoming increasingly important as the quantity of data available to farmers and land managers continue to increase, the availability of this data in conjunction with the facilities allows our knowledge exchange advisors to disseminate the results of these studies widely and with impact on business across Scotland and the rest of Europe. This will help rangeland land managers in achieving Net-Zero greenhouse gas emissions with greater emphasis on applying nutrients more effectively to lowland grasslands, tracking and monitoring livestock location and health, and restoring degraded peatlands where feasible (McCracken, 2021). Access to real-time data to aid management decision-making will also be helpful in managing Scotland’s rangeland areas in ways that mitigate or prevent flooding further downstream in water catchments. Given that much of Scotland is remote and challenging topographically, the use of sensors together with improved connectivity and processing power to collect, transmit and process such data is essential. The lessons learnt from this Hub will also be applicable to much of the other rangeland areas in the world (Sm@RT & DIGIRangeland projects), and similar work carried out on other UK farm platforms (e.g. the North Wyke Farm Platform) (Orr et al., 2016) will also contribute to increase the awareness and knowledge regarding those issues.

Using the available data, the idea is to move toward a *Digital Twin* with our digital hub architecture, to rapidly test possible land use models and approaches *in silico* before ground truthing them on the farm itself (Blair and Henrys, 2023). Furthermore, the availability of such a concept would allow knowledge exchange advisors to demonstrate to land managers how they could implement similar systems on their own land and allows provision of ‘live’ datasets direct to learners, helping both to train the next generation of innovative land managers and also upskill and reskill those currently in employment.

Moving toward a Digital Twin concept would be fundamental to helping monitor and assess some of the research projects carried out on the Rangeland Digital Hub (Table 1).

**Table 1.** Approaches and outputs of the themes covered within the Rangeland Digital Hub

Themes	Projects and approaches	Outputs
A Rangeland Digital Hub	Sm@RT Small Ruminant Technologies Multi-actor workshops to identify needs and solutions relating to the uptake of technologies in small ruminant systems. Use of Digifarms as demonstration places (Kirkton & Auchtertyre farm was one of the DigiFarms)	List of farmers’ needs for technological solutions* List of technological solutions (with guidelines and videos) relevant to the extensive grazing systems
	DiGiRangeland www.digirangeland.eu Multi-actor workshops to identify needs and digital solutions for rangeland areas Use of Innovation & Demonstration Hubs—Kirkton & Auchtertyre farm is one of these ID-HUBs)	List of rangeland stakeholders’ needs for digital solutions List of digital solutions relevant to stakeholders in rangeland areas
Developing rangeland livestock systems fit for the 21st century	Breeding and management strategies for sheep in the Scottish hills and uplands, to meet future economic, environmental & climatic challenges (https://sefari.scot/research); GrasstoGas (https://www.eragas.eu/grasstogas; Sustain Sheep (https://www.greenerahub.eu/sustain-sheep) LUNZ Grassland (https://lunzhub.com/projects/grasslands/) Genetic selection Measurement of novel phenotypes using: • Portable Accumulation Chambers • Computerized Tomography scanning • Feed intake recording • Digitanimal GPS collars	Grazing homerange of individual ewes Methane emissions from individual ewes & lambs**^$^** Feed intake & efficiency of individual lambs**^$^** Body composition and rumen volume of ewes & lambs Phenotypic and genetic relationships among traits
	Cattle hill grazing & Conservation grazing Use of virtual fencing to monitor hill grazing**^&^**	Most appropriate cattle grazing densities and wintering strategies
Monitoring welfare and performance in small ruminant systems	TechCare www.techcare-project.eu Multi-actor approach (with regular stakeholders’ workshops) to decide which technological innovations could help monitor identified welfare issues**^#^** Technological innovations are tested on pilot farms (Kirkton is one of them) alongside welfare assessments^+^,^=^	List of welfare issues (and their assessment protocol) relevant to extensive sheep grazing systems. List of existing tools and promising prototypes for extensive grazing systems [EID weigh crate, Bluetooth beacons and readers, RFID (LF & UHF) readers and tags, connected weather stations]
Developing natural capital assessment metrics	Seeking multiple benefits from natural carbon stores in the uplands (https://sefari.scot/research/projects/seeking-multiple-benefits-from-natural-carbon-stores-in-the-uplands) Use of digital audio devices in conjunction with machine learning software to automatically identify birds and bats. Use of baited camera trap boxes to monitor small mammal activity Use of Long Range Wide Area Network (LoRaWAN) sensors to monitor river levels, rainfall, soil moisture, air temperature etc.	Guidance on the use of audio digital devices for biodiversity monitoring**^£^** Key biodiversity, carbon and flood mitigation metrics for the Scottish uplands**^£^**

* McLaren et al. (2022).

$ Le Graverand et al. (2025).

& Waterhouse (2023).

# Morgan-Davies et al. (2024).

+ Waterhouse et al. (2023).

= McLaren et al. (2025).

£ Holland et al. (2025).

### Developing rangeland livestock systems fit for the 21st century

Agricultural land use in rangeland Scotland is dominated by rough grazing (mountain pasture) and marginal grassland systems. Much of this land is grazed by sheep, a key industry for Scotland, and rangeland sheep production is at the heart of many challenges related to land use, greenhouse gases (GHG) emissions, habitat conservation, and ecosystems services. Income from rangeland sheep businesses is low and change is required to improve productivity of this key agricultural land use type. One aim of the Rangeland Digital Hub is to produce a composite sheep breed for harsh Scottish mountain environments, which not only has a diverse genetic base suitable to cope with productivity challenges relating to climate change but also emits less greenhouse gases and produces a carcass desired by processors and consumers. Hence, the program of work is combining state-of-the art phenotyping for efficiency and resilience traits with genetic selection, utilizing innovative feed intake recording bins and Computerized Tomography (CT) scanning on-site (to assess actual efficiencies and carcass quality achieved) and assessment of methane emissions from individuals through use of Portable Accumulation Chambers (PACs). Participation in a range of international collaborations (see Table 1) not only further enhances our research capabilities at the farm but will also reinforce the role of the Rangeland Digital Hub in shaping the future of global sustainable livestock production.

Historically cattle were an important feature on rangeland farms, with mixed grazing by sheep and cattle of mountain pastures not only helping to improve overall livestock productivity but also maintain biodiversity by encouraging a greater variety of plant species in mountain swards. Production driven economics and labor constraints have resulted in a marked decline in cattle in these systems, particularly in the Highlands & Islands of Scotland. However, the increasing recognition of mixed livestock grazing as a nature conservation tool (Fraser et al., 2014) coupled with rising consumer interest in more naturally reared beef, means there is a need to reconsider the current sheep to cattle ratio on rangeland farms. This will not only require a focus on the most appropriate cattle grazing densities and wintering strategies to employ but also the use of innovative technologies (such as virtual fencing) to track and manage cattle while out grazing the open rangelands.

### An example of digital innovation use: monitoring welfare and performance in small ruminant systems

Although these livestock systems, particularly sheep systems, play a key socio-economic role in Scotland and in Europe, innovative technology is not widely implemented, and the animals are often only managed at flock level, allowing only average welfare states and performance to be considered. Innovative technologies are a unique opportunity to monitor and improve sheep welfare management both at the individual or flock level (Morgan-Davies et al., 2024). One of the projects (TechCare—www.techcare-project.eu) carried out on the Rangeland Digital Hub has showcased how a multi-actor approach to encapsulate stakeholders’ expectations in terms of welfare and innovative technologies could provide adapted solutions and novel welfare approaches. These approaches aimed to develop potential alerts (e.g. mismothering, loss of condition, animal loss) for sheep farmers. Both existing technologies [such as Radio-Frequency Identification (RFID) Electronic Identification (EID)] and prototypes have been tested and validated. Individual weight monitoring (Morgan-Davies et al., 2018), proximity to feed resources (Waterhouse et al., 2023) and ewe-lamb distances to identify mismothering issues (Walker, 2025), to name a few, have been studied and showed promising results using these affordable digital innovations. Data exchanges and retrieval from the various sensors deployed was made possible using the IoT network and Digital Hub capacity.

### Integrating greater environmental management into rangeland farms

As already highlighted above, rangeland farmers also need to take action to sequester more carbon on their land if Scotland (and Europe) is to achieve the target of net zero emissions. Over 200 ha of montane woodland was established in one of the valleys in this Rangeland Digital Hub 27 years ago and around 100 ha of degraded peatlands were restored 10 years ago. However, simply restoring peatland or planting new woodland does not in itself result in immediate carbon sequestration benefits, as these potentially will take years to be achieved. In addition, management of the rangelands of Scotland will have an important role to play in mitigating flooding events further downstream by managing water quantity, and it is essential to assess what additional management is needed—at what scale and where—to best help achieve this. The aim for the Rangeland Digital Hub is to demonstrate how small areas of more productive woodland—both in small blocks and within fields as agroforestry—can be established on the lower parts of the farm without interfering with agricultural management (McCracken, 2024a). Working with the local Tay District Salmon Fisheries Board and Loch Lomond & The Trossachs National Park, the Hub is also seeking to demonstrate the benefits of managed retreat, by breaching one part of the existing river embankment to provide for more storage of water on the farm during extreme flood events (monitored using environmental sensors—Figure 3). The need to assess the agricultural and environmental implications over time once the embankment has been breached is being emphasized in those discussions.

### Increasing the socio-ecological viability of rangeland farming

As highlighted previously, many of Scotland’s rangeland farms are considered to be of High Nature Value (HNV) in that they are rich in natural and semi-natural vegetation. They support species and habitats of important conservation value which rely on farm management practices for their maintenance. In addition to providing humans with food and fiber, HNV farmland also supports biodiversity conservation and delivers a wide range of vital public services such as managing flood risk, protecting soils from erosion, or having cultural value. However, these HNV farmlands are currently economically unviable, and abandonment of farming practices is leading to an erosion of their natural, social and cultural heritage. Reversing the decline of HNV farmlands relies on increasing public appreciation of the range of goods and services provided by these landscapes and improving the financial rewards to farmers who continue to maintain them. A paradigm shift is required to ensure that elements of the underlying systems persist and that HNV systems appeal to future generations (Lomba et al., 2020). Ongoing work on the Rangeland Digital Hub allows demonstration of how such a shift in practice entails moving away from current production-driven financial incentives to farmers, toward novel incentive mechanisms rewarding a broader range of goods and services and integrated landscape-level approaches where direct links between people and nature are fostered to build economic, social, and environmental sustainability (McCracken, 2022a). In particular, the future of HNV farmlands is likely only to be secured through a combination of improving social services in rural communities, designing new uses for HNV goods, and developing new business opportunities on HNV farmlands. Digital innovation is also part of that vision.

### Developing natural capital assessment metrics

Natural capital is recognized as a significant and yet undervalued resource in rangeland areas, with great potential to transform and support rural businesses and regions that possess an abundance of natural resources. However, although payments for natural capital management and enhancement are increasingly being suggested, the lack of transparent, robust and easily repeatable metrics is limiting the ability to develop such payment approaches (McCracken, 2022b). Without such metrics, any potential public or private investor remains uncertain as to whether proposed outcomes are either achievable or deliverable. Using environmental sensors as part of a panel of tools, metrics applicable to the upland setting (such as the amount of carbon stored in soils and woodlands, the amount and condition of different habitats and the role they play in holding back water and supporting a wide range of biodiversity) will be developed and tested on the Rangeland Digital Hub, as well as on other similar characteristic habitats within the headwaters of the River Tay (McCracken, 2024b).

## Conclusions—Delivering a Digital Vision with Knowledge Exchange for Impact

Over the last 13 years, the number of visitors to the farm (between 30,000 to 50,000 people passing through the farm), together with events (over 40 per year on average) being held at the farm, have increased markedly. There is scope to do more, to explore fully a Rangeland Digital Hub approach. Increasing wider skills training that occurs on site, increasing use of the farm by students from across Scotland and further afield for practical activities associated with future approaches to agricultural and environmental management, are key aspirations. Being involved with the *Wild Strathfillan Initiative* (https://trustinthepark.org/wildstrathfillan/) and *Our Rainforest Futures* (https://www.communitywoods.org/our-rainforest-futures-project) projects (to name a few) is one way of increasing the use of the hub for such practical training. The improved instrumentation of the site also lends itself to enhanced knowledge exchange, particularly as knowledge exchange increasingly takes on a mixed mode including digital and face to face approaches. Previous (e.g. SheepNet, Eurosheep) and ongoing European funded network projects have and are focused on knowledge transfer and exchange of best practices between farmers and other stakeholders from many European countries (see Table 1), and Kirkton & Auchtertyre farm has been the epicenter of these networking activities and training (both a Digifarm and DiGi-Hub).

Ongoing relationship and further exchanges with the other farm platforms in the UK and in the Global Farm Platform Initiative will add to the wealth of information being tested and shared, to truly deliver a digital vision for rangeland areas.

### Final words—global relevance of our digital vision for rangeland grazing systems

Over one third of the world’s land area consists of rangelands where pastoralists graze their animals on natural or semi-natural habitats. Hence, the research at Kirkton & Auchtertyre has global relevance and we intend to continue to work on projects involving a wide range of partners from across Europe and the globe and to build upon our membership of the Global Farm Platform Initiative. The elements of agricultural, environmental and natural capital research and demonstration highlighted above also mean that we are well placed to establish the farm as a Living Lab and proper farm platform of relevance to both national and international partners and collaborators.

## Author Biographies

Dr. Claire Morgan-Davies is Reader in Livestock Systems at Scotland’s Rural College (SRUC). Claire is based at SRUC’s Hill and Mountain Research Centre near Crianlarich, where she has focused on participatory research, knowledge exchange, and research on extensive sheep systems for over 25 years. In the past 10 years, her research has focused on technology use, uptake and adoption by small ruminant farmers in the UK and Europe.

Dr. Nicola Lambe has around 30 years’ experience working on sheep research projects, investigating improvements in product quality, production, and reproductive traits, in different sheep systems and breeds. She is a Reader, based at SRUC’s Hill and Mountain Research Centre and manages the SRUC GreenSheep facilities, including CT scanner, portable accumulation chambers, and feed intake recording equipment. These facilities quantify a variety of new sustainability traits for use in research and commercial sheep breeding. Her recent projects have focused on measurement of, and genetic selection for, traits including methane emissions and feed efficiency.

Dr. John Holland is an upland ecologist at Scotland’s Rural College (SRUC). John is based at SRUC’s Hill and Mountain Research Centre in highland Perthshire, where he has carried out biodiversity and environmental research and knowledge exchange for over 30 years. His current research interests focus on biodiversity and ecosystem services in the uplands, the grazing behaviour of sheep, and the use of technology in environmental, biodiversity, and livestock monitoring.

Dr. Ann McLaren is a researcher at Scotland’s Rural College (SRUC). Ann has been based at SRUC’s Hill and Mountain Research Centre for over 20 years, where her interests have focused on different aspects of sheep productions systems. The main themes of her research include sheep breeding programs and investigating the genetic aspects of new and novel traits, plus the use and uptake of technology in sheep systems.

Professor Davy McCracken joined SRUC: Scotland’s Rural College in 1995 and has been Head of SRUC’s Hill & Mountain Research Centre, at Kirkton & Auchtertyre farms near Crianlarich, since 2013 and Deputy Head of the School of Natural & Social Sciences since 2025. The farms at Crianlarich are a *Linking Environment & Farming (LEAF) Innovation Centre* and a member of the *Global Farm Platform* initiative. Consequently, the research and demonstration on the farms have a particular focus on how precision livestock approaches and associated technologies can be used to address agricultural and environmental challenges in the uplands. Davy studies farming and wildlife interactions and has been working on agricultural and agri-environmental policy at a national and international level for over 30 years. He was one of the founders of the European High Nature Value farming concept. Davy has been involved in providing advice and guidance on policy development to a wide range of governmental and NGO committees over the years and is currently inputting to the development of both Scotland’s new agricultural support policy and future biodiversity strategy.
